# Effect of different landing actions on knee joint biomechanics of female college athletes: Based on opensim simulation

**DOI:** 10.3389/fbioe.2022.899799

**Published:** 2022-10-26

**Authors:** Liang Chen, Ziang Jiang, Chen Yang, Rongshan Cheng, Size Zheng, Jingguang Qian

**Affiliations:** ^1^ Department of Sports Science, Nanjing Sport Institute, Nanjing, China; ^2^ Department of Orthopaedic Surgery, Shanghai Ninth People’s Hospital, Shanghai Jiao Tong University School of Medicine, Shanghai, China; ^3^ School of Biomedical Engineering and Med-X Research Institute, Shanghai Jiao Tong University, Shanghai, China; ^4^ School of Mechanical Engineering, Zhejiang University, Hangzhou, China

**Keywords:** landing test movements, ACL, opensim, knee joint, biomechanics

## Abstract

**Background:** The anterior cruciate ligament (ACL) is one of the most injurious parts of the knee in the biomechanical environment during landing actions. The purpose of this study was to compare the lower limb differences in movement patterns, muscle forces and ACL forces during drop landing (DL), drop vertical jump (DVJ) and forward vertical jump (FVJ).

**Methods:** Eleven basketball and volleyball female college athletes (Division II and I) were recruited. Landing actions of DL, DVJ and FVJ, kinematics and dynamics data were collected synchronously using a motion capture system. OpenSim was used to calculate the ACL load, knee joint angle and moment, and muscle force.

**Results:** At initial contact, different landing movements influenced knee flexion angle; DL action was significantly less than FVJ action (*p* = 0.046). Different landing actions affected quadriceps femoris forces; FVJ was significantly greater than DL and DVJ actions (*p* = 0.002 and *p* = 0.037, respectively). However, different landing movements had no significant effects on other variables (knee extension moment, knee valgus angle and moment, hamstring and gastrocnemius muscle forces, and ACL forces) (*p* > 0.050).

**Conclusion:** There was no significant difference in the knee valgus, knee valgus moment, and the ACL forces between the three landing actions. However, knee flexion angle, knee extension moments sagittal factors, and quadriceps and gastrocnemius forces are critical factors for ACL injury. The DL action had a significantly smaller knee flexion angle, which may increase the risk of ACL injury, and not recommended to assess the risk of ACL injuries. The FVJ action had a larger knee flexion angle and higher quadriceps femoris forces that were more in line with daily training and competition needs. Therefore, it is recommended to use FVJ action in future studies on risk assessment of ACL injuries and injury prevention in female college athletes.

## Introduction

The anterior cruciate ligament (ACL) injuries in female athletes are four to six times higher compared to male athletes (Agel et al., 2005). Mainly, ACL injuries are classified as non-contact injuries and usually occur in landing, plant-and-cut, and twisting actions (Meyer and Haut, 2008). Landing is one of the everyday sports actions in basketball, volleyball, and other sports activities (Fort-Vanmeerhaeghe et al., 2016). The previous studies reported that overly small knee or hip flexion, overly large knee valgus angle, extension moment, the valgus moment might be leading causes of ACL injuries (Chappell et al., 2002; Kernozek et al., 2005). Further, the muscle reaction forces such as excessively high quadriceps muscle force and low hamstring muscle contraction force can also contribute to the ACL injury during landing in professional female athletes.

Currently, the landing test actions in risk assessment of ACL injury mainly include drop landing (DL) (Decker et al., 2003; Kernozek et al., 2005), drop vertical jump (DVJ) (Cortes et al., 2007), and forward vertical jump (FVJ) (Padua et al., 2004). Wenxin et al. (Niu et al., 2013) reported the dynamic postural stability and the influence mechanism of muscle activities during DL from 30cm, 52cm, and 72 cm. Satoshi et al. reported the effects of multi-task interference on the biomechanics of the lower limb during the DVJ while Hossein et al. compared the lower limb differences in movement patterns, muscle forces, and ACL forces during DVJ (Mokhtarzadeh et al., 2017; Imai et al., 2022). Padua et al. (2009) use FVJ as a standard action for Landing Error Scoring System (LESS) testing and treated it as one of the most efficient ones for ACL injury risk tests. These tasks are functionally relevant to athletic performance and execution of these complex skills requires well-trained and coordinated movement. However, most previous studies used only one test protocol, which also led to some differences in the findings of the different studies, which may be related to the different task requirements of the test movements, thus changing the human movement pattern. In addition, these studies extensively used the inverse dynamics method to qualitatively and quantitatively determine the risk factors of ACL injury during exercise (Bennett et al., 2008; Padua et al., 2009; Blackburn et al., 2013; Mokhtarzadeh et al., 2017; Bulat et al., 2019; Imai et al., 2022). Although the calculation process and experimental method are unified, significant divergence still exists in the estimated ACL load (Bennett et al., 2008; Blackburn et al., 2013) due to limitations in the experimental object and the algorithm. Blackburn et al. (2013) calculated the peak value of anterior tibial shear force during DL action (30 cm height) through inverse dynamics, and it was about 0.7BW, while the result of Bennett et al. (2008) reported only about 0.4BW under the same method and landing action. However, some studies demonstrated that muscle forces are one of the main determinants of the dynamic load of the knee ligaments, and the ligaments balance the anterior tibial shear force during landing (Dürselen et al., 1995; Anderson and Pandy, 2003; Bennett et al., 2008; Blackburn et al., 2013). The quadriceps and hamstring muscle groups make function during landing is critical in determining the load experienced by the knee joint structures and therefore fundamental to understanding ACL injuries. Therefore, estimating the ACL load during landing by calculating anterior tibial shear forces through the inverse dynamic method has limitations affecting the accurate evaluation of the risk factors of ACL injury.

The simulation technology plays an essential role in complex biomechanical problems (Reinbolt et al., 2011), and it can simulate the mechanisms of the musculoskeletal system during human motion through an optimization framework of forward and reverse analysis (Rajagopal et al., 2016). Many studies have demonstrated that the simulation optimization method of the OpenSim musculoskeletal numerical simulation system is superior to the traditional inverse dynamics or mathematical methods (Delp et al., 2007; Seth et al., 2018; Yu et al., 2020). Recently, Huiyu et al. reported the effects of different footwear on lower limb kinematics, kinetics, and muscle forces in walking and running movements by OpenSim (Zhou et al., 2021). It is also considered one of the most widely used software for kinematic and dynamic analysis in complex actions such as running, jumping, and landing (Kar and Quesada, 2012; Mokhtarzadeh et al., 2017; Zhou et al., 2021; Renganathan, 2022), while providing validated platforms for exploring the nerve-muscle performance in dynamic activities. Although there are studies that assessed the risk of ACL injury under landing test actions in female athletes (Decker et al., 2003; Kernozek et al., 2005), there are no reports on quantified ACL loads in parallel with the three landing actions (DL, DVJ and FVJ). This research explored the differences and relationships of ACL load, knee angle, joint moment, and muscle forces in three landing test actions of professional female athletes using the OpenSim system. Further, it provided a theoretical basis for selecting appropriate landing assessment movements and ACL injury prevention.

Accordingly, we hypothesize as follows: 1) the differences in the knee angle, moment, and muscle forces of the three test actions. 2) ACL injury had a higher risk for DL action than for DVJ and FVJ.

## Materials and methods

### Subjects selection

The sample size was calculated according to a previous study protocol (Decker et al., 2003; Fagenbaum and Darling, 2003; Cortes et al., 2007). Eleven female athletes (4 division I basketball players and 7 division II volleyball players) from the Nanjing Sports Institute were recruited (age: 21.7 ± 0.7 years; height: 171.5 ± 4.6 cm; weight: 63.1 ± 9.0 kg). The inclusion criteria were no history of lower limb injury and not engaged in strenuous exercise within 48 h before the experiment. All the participants had greater than 10 years’ sufficient training and competition experience on sports. Subjects have all given written informed consent for participation and were familiar with the experiment process.

### Experiment process

**FIGURE 1:**
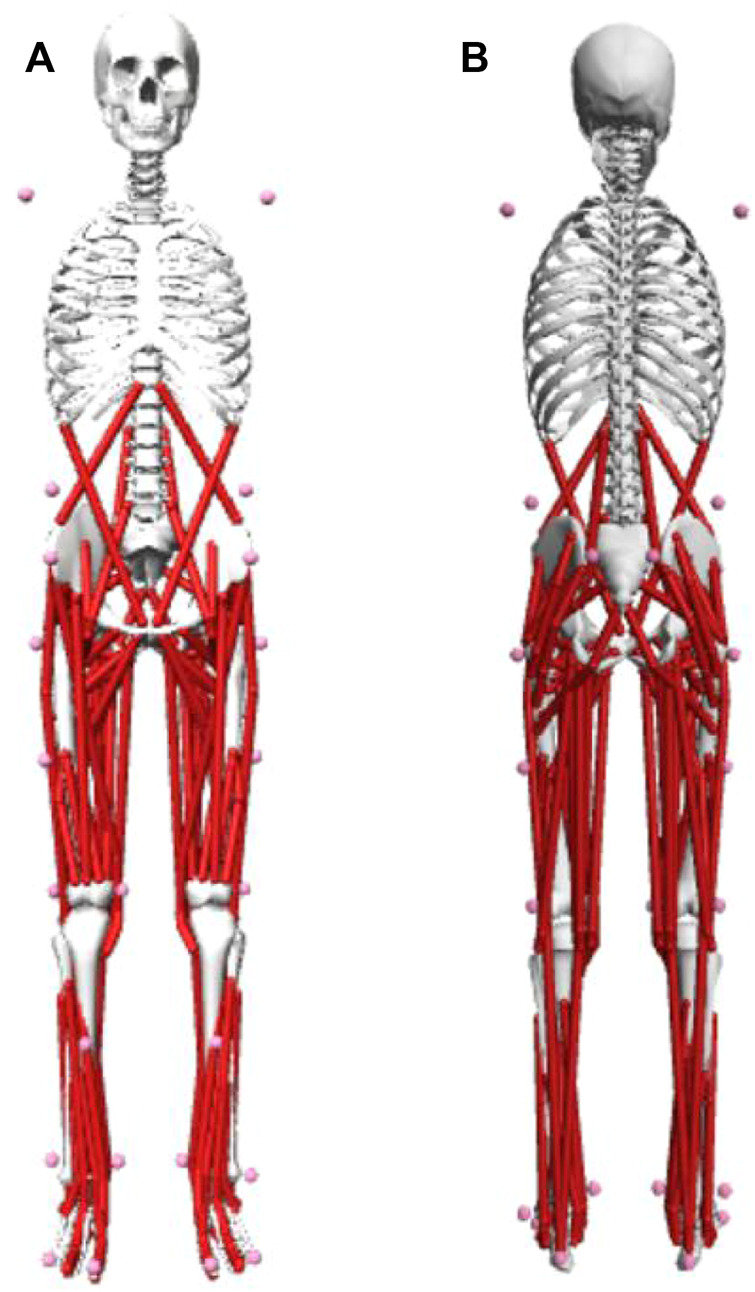
Schematic representation of the placements of the marker. **(A)** The front vision of the marker set; **(B)** the back vision of the marker set.

Twenty-eight 14 mm infrared-reflective markers were firmly secured on each participant to characterize the anatomical bone landmarks. Bone landmarks included acromion, the highest point of the iliac spine, anterior superior iliac spine, posterior superior iliac spine, greater trochanter, medial and lateral femoral condyle, medial and lateral malleolus, heel, the first and the fifth metatarsal, as shown in Figure 1. The markers were pasted on bilateral bone landmarks to ensure the complete capture of the participants’ actions. Four additional markers were secured in the middle of the thigh and shank bilaterally to prevent the loss of a marker while capturing. An experienced researcher manually located anatomical bone landmarks through the skin surface and pasted each marker. The methodology of pasting markers was primarily based on the study of Kar and Quesada (2012). However, certain modifications were done according to the modelling coordinate points guide in Visual3D inverse dynamics software (Visual3D, C-Motion, Inc., United States).

The trajectories of markers were captured during the landing process by 12 Vicon motion capture cameras (200 Hz, Vicon Motion Analysis Inc., Vantage 5, Oxford, United Kingdom). Two fully integrated three-dimensional force platforms (1000 Hz, AMTI, Watertown, Massachusetts, United States) synchronously captured the dynamic ground-reaction force signal with the motion capture system. Surface electromyography (EMG) data were collected using a Telemetered system, was used to collect muscle activations. The surface electrodes were applied to the participant’s dominant limb over the muscle bellies of the rectus femoris, vastus lateralis, biceps femoris, and tibialis posterior.

**FIGURE 2:**
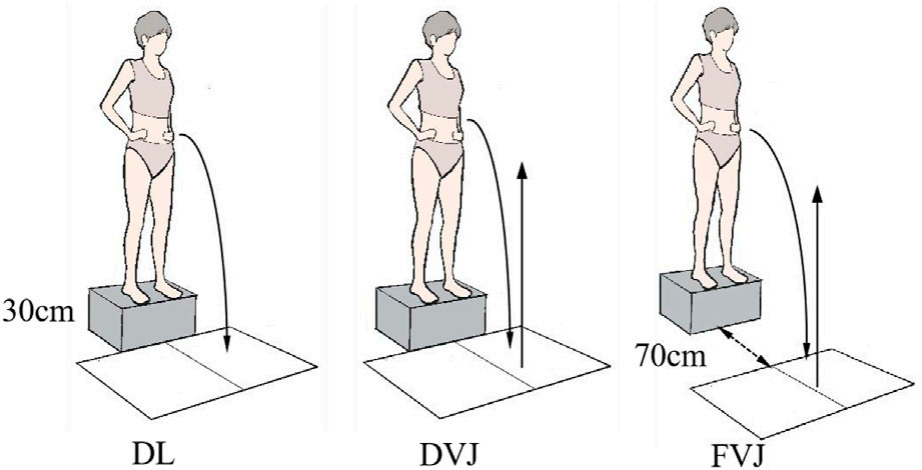
DL, DVJ and FVJ landing test action.

Before the experiment, each participant was well instructed on the movement performance for the assessment, instructions and cadence which were standardized according to the experimental protocol (Decker et al., 2003; Padua et al., 2004; Kernozek et al., 2005; Cortes et al., 2007). Participants completed the following tasks in sequence: (1) DL: the participants were instructed to jump forward from a block 30 cm high with arms akimbo and land with their feet on two separate force platforms. (2) DVJ: the take-off process was similar to DL. After the first landing, participants were instructed to perform a maximal vertical jump coherently. (3) FVJ: the block was 70 cm away from the force platform. Participants were instructed to land on the center of the force platform and perform a maximal vertical jump coherently after the first landing (Figure 2). All the subjects were asked to warm up in each self-select activities before the experiment started and practiced the three landing actions until be familiar with the test movements and process. The formal experiment was preceded and followed by a 5-min rest period.

Participants performed the three landing actions until three valid trials were recorded, and the final result was based on the average of three valid trials. A trial was acceptable when the participant jumped from the block without falling due to loss of balance. Further, the trial was invalid if their hands did not remain akimbo or the feet did not land separately on two different force platforms.

### Primary data process

The kinematics and dynamics data of the trials were filtered using a low-pass filter (Butterworth 4th order) and run with a cutoff frequency of 10 Hz for the motion capture system and 100 Hz for force signal in the inverse dynamics software, Visual3D (Koltermann et al., 2018).

Firstly, a 4th-order band-pass filter between 10 and 450 Hz was applied to the EMG data, before it was full-wave-rectified. Finally, the Root Mean Square RMS was calculated. The data from the initial contact (IC) phase to the maximum knee flexion (MKF) phase were selected for processing. The data from the dominant side of the athletes were analyzed in three landing test actions. Knee kinematics, dynamics, and muscle forces data of the three landing actions were temporally standardized in Origin 2019b software. Raw data were interpolated to 101 data points, and 0% represented the touching moment, and 100% represented the maximum flexion moment of the knee joint. The direction of the joint angle and moment was defined as positive sagittal knee extension; frontal knee valgus is positive.

### Statistical analysis

The biomechanical parameters of the lower limbs from three landing test actions were analyzed in SPSS (Version 20.0., IBM Corp., Armonk, NY, United States). Data were tested for normality and equal variance before the analysis. If the data met normal distribution assumptions and equal variances, analysis of variance was used (one-way ANOVA), and nonparametric Kruskal–Wallis test was used when the variance was not homogeneous. The post-hoc comparison was performed by the least significant difference (LSD) test of mean values. The effect size is represented by a partial eta square (η^2^), and *p*-value < 0.05 was considered significant. Statistical Para-metric Mapping (SPM) was also applied in the comparison of the kinematic and dynamic result in the process of three actions performance which was a one-dimensional methodology for the topological analysis of smooth continuum changes associated with experimental intervention (Pataky, 2012). The significance level of the SPM{F} characteristics of the test for joint angles, joint moments, and muscle forces was set at α = 0.05, while F statistics are considered as the aforementioned family of linear statistical tests. All the Statistical analysis was performed in MALTLAB (MATLAB R2018a, The MathWorks, MA, United States).

### Biomechanical data analysis

**FIGURE 3:**
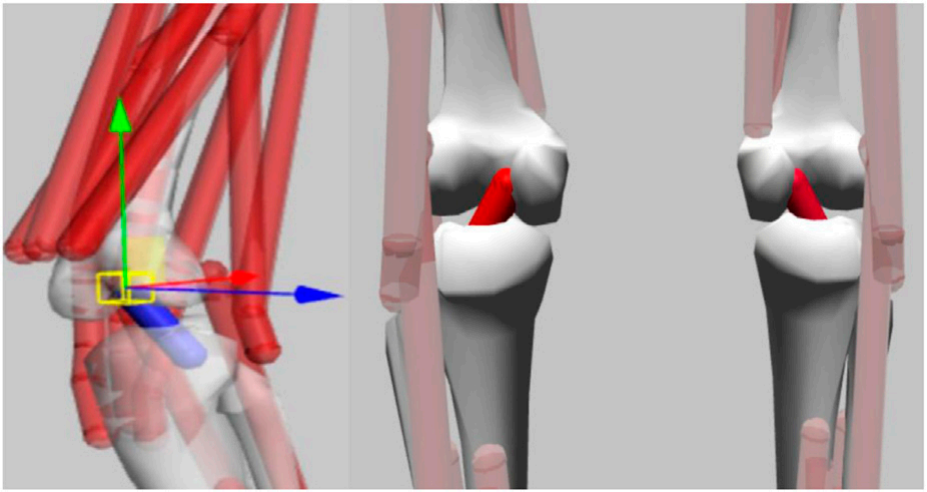
Gait2392 model with ACL model imported in OpenSim.

This study used a simplified generic Gait2392 musculoskeletal model in OpenSim (OpenSim 4.1, Stanford, California, United States), with 12 bony segments, 92 muscles, and 23 degrees of freedom (Kainz et al., 2016). This study mainly focused on the ACL load and the variation between the knee joint variables in three landing test actions. Therefore, the ACL model was introduced into the original Gait2392 model, and the degree of freedom (DOF) on the frontal plane of the knee joint was also added. The generalized coordinates, physiological parameters and the DOF function of ACL were referred to the verified results (Kar and Quesada, 2012; Kar and Quesada, 2013) (Figure 3).

**FIGURE 4:**
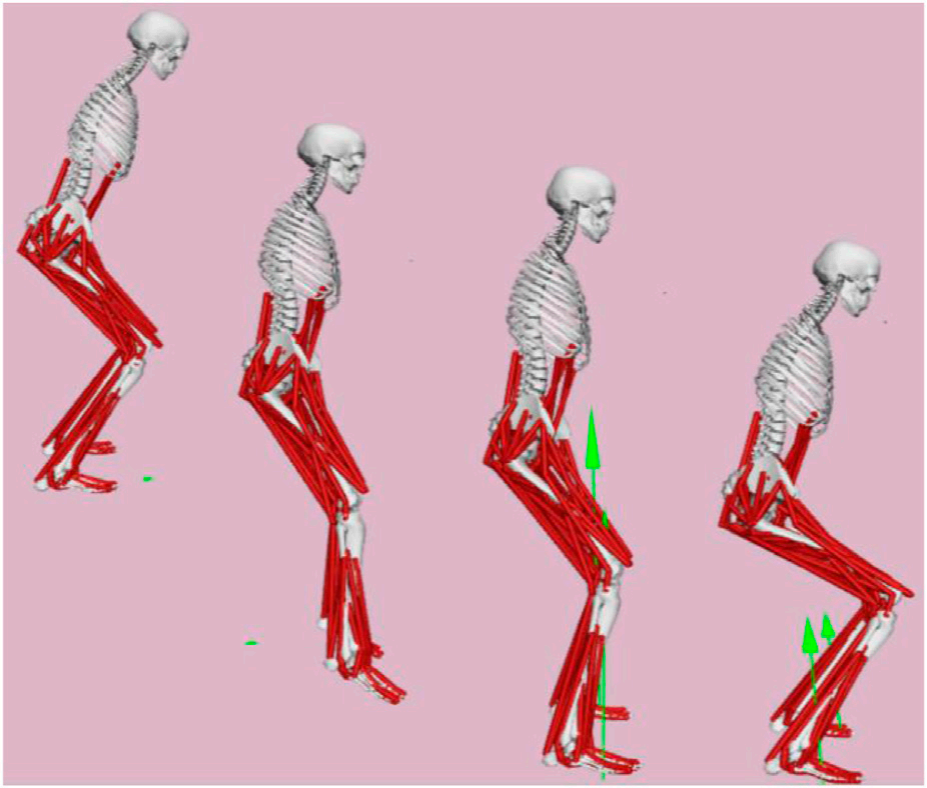
Schematic of the OpenSim simulation results. Note: Since the main bearing stage of ACL during landing occurs from the IC to MKF, the simulation results of the three landing test actions in this study were taken during the IC-MKF (0%–100%) stage (Panel).

The musculoskeletal model was scaled (Scaling) to generate the real anthropometry, and the Inverse Kinematics (IK) calculation module from the Visual3D was input into the OpenSim. The inverse simulation of the three landing actions was achieved through the OpenSim Residual Reduction Algorithms (RRA), Computer Muscle Control (CMC), and Forward Dynamics (FD) tool (Figure 4). The dependent variables such as the knee biomechanical parameters, muscle forces and ACL load were calculated while time was used as the independent variable.

The muscle forces calculated in our study are as follows: Quadriceps femoris (rectus femoris, vastus lateralis, vastus medialis and vastus intermedius), Hamstring (biceps femoris lh, biceps femoris sh, semitendinosus and semimembranosus), Gastrocnemius (medial, gastrocnemius, lateral gastrocnemius and soleus).

## Results

**FIGURE 5:**
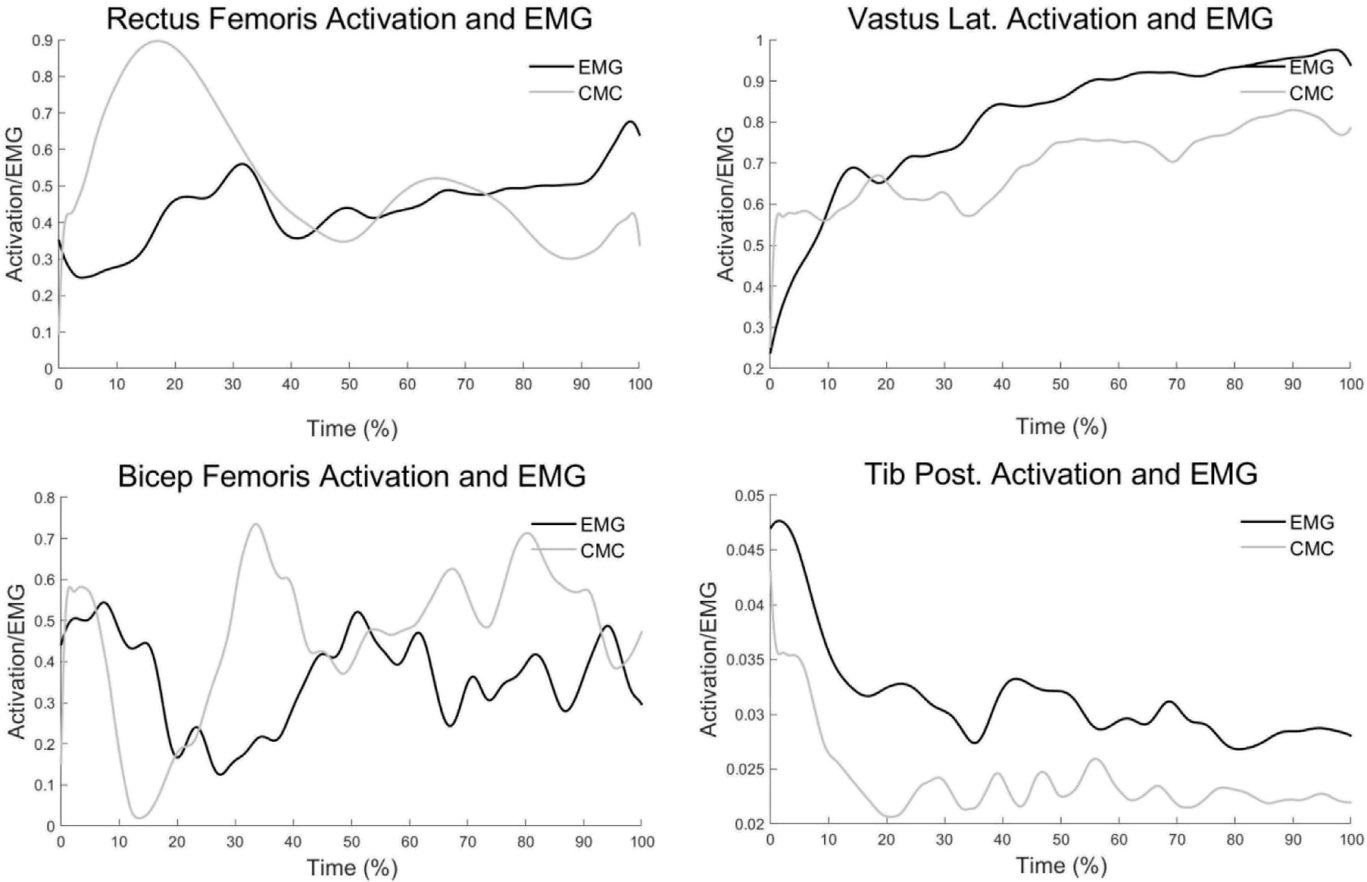
Comparisons between EMG signal and muscle activation estimated from CMC in OpenSim.

**FIGURE 6:**
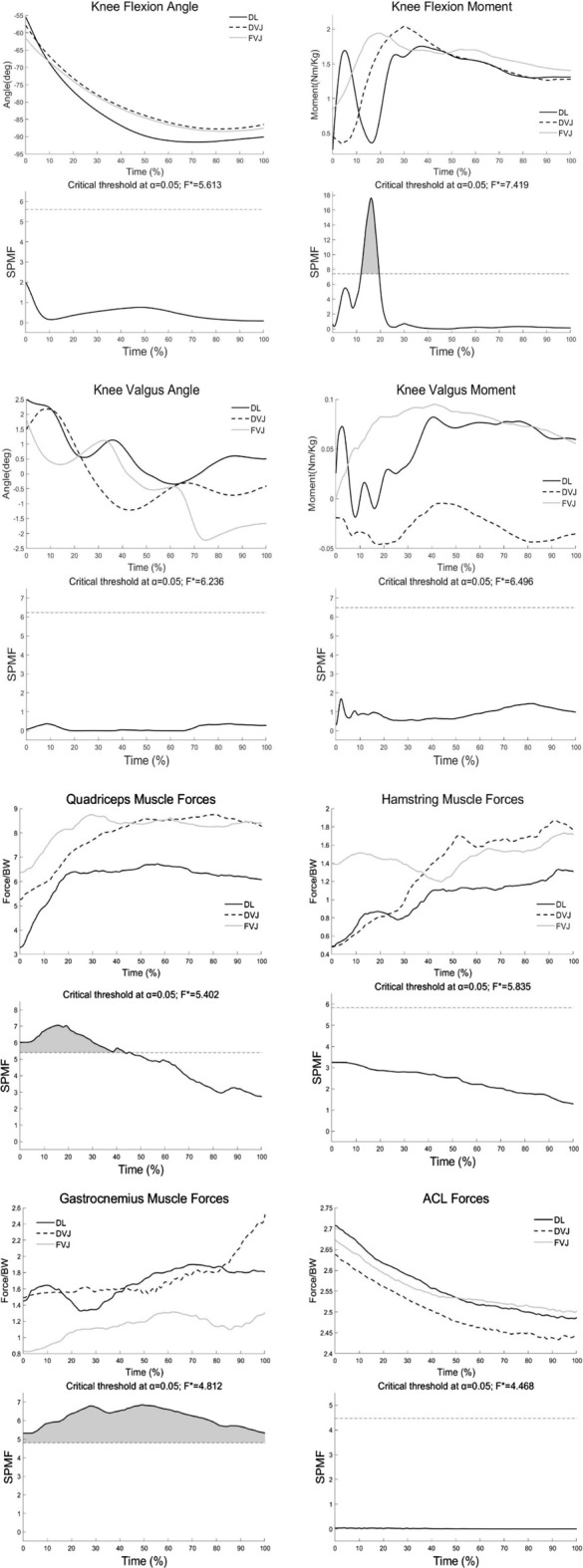
Ensemble average and SPM{F} evaluation of right knee variables against percentage time obtained from 11 subjects participating in the three landing actions.

As shown in Figure 5, Calculated four muscle activations and their corresponding measured EMGs were also in good agreement qualitatively. As shown in Table 1 and Figure 6, One-way ANOVA showed that different landing actions had significant effects on the knee flexion angle at IC (*p* = 0.046). A subsequent test showed that the knee flexion angle of the DL action was less than the FVJ action. One-way ANOVA showed that different landing movements had affected quadriceps femoris forces at IC. Secondary tests showed that the quadriceps femoris forces under the FVJ action were significantly greater than the DL and DVJ actions (*p* = 0.002 and *p* = 0.037, respectively). Knee extension moment, knee valgus angle, knee valgus moment, hamstring muscle forces, gastrocnemius muscle forces, and ACL forces had no significant effects (*p* > 0.05).

**TABLE 1:** Statistics of knee variables from the IC phase of three landing actions performed by 11 subjects.

Variables	Mean ± SD_DL	Mean ± SD_DVJ	Mean ± SD_FVJ	*p*-value	η^2^
Knee flexion angle (^o^)^c*^	−55.56 ± 7.23	−57.91 ± 7.58	−61.77 ± 4.53	0.05	0.14
Knee flexion moment (N/kg)	0.26 ± 0.32	0.45 ± 0.99	0.84 ± 1.58	0.87	0.06
Knee valgus angle (^o^)	2.49 ± 4.48	1.51 ± 3.82	1.81 ± 4.00	0.85	0.01
Knee valgus moment (N/kg)	0.03 ± 0.06	−0.02 ± 0.11	0.00 ± 0.17	0.81	0.03
Quadriceps femoris forces (N/BW)^a*c*^	3.29 ± 1.90	5.27 ± 1.38	6.37 ± 2.58	0.00	0.31
Hamstring muscle forces (N/BW)	0.49 ± 0.43	0.49 ± 0.69	1.40 ± 1.48	0.26	0.18
Gastrocnemius muscle forces (N/BW)	1.45 ± 0.45	1.50 ± 0.35	0.83 ± 0.67	0.28	0.28
ACL forces (N/BW)	2.71 ± 0.37	2.64 ± 0.40	2.67 ± 0.36	0.44	0.01

(1) a* indicates a significant difference between DL, and DVJ; b* indicates a significant difference between DVJ, and FVJ; and c* indicates a significant difference between DL, and FVJ. (2) The unit of joint moment data was N/kg which was standardized according to the body mass (kg). The muscle forces and ACL, load were standardized in N/BW, according to body weight (BW). (3) While η^2^ = 0.01 is a small effect, η^2^ = 0.06 is a medium effect, and η^2^ = 0.14 is a large effect.

As shown in Figure 6, The SPM{F} showed that different landing actions had significant effects on the knee flexion moment at IC-MKF from 11% to 21% (α = 0.05, F = *7.42). The SPM{F} showed that different landing actions had significant effects on the quadriceps femoris forces at IC-MKF from 0% to 41% (α = 0.05, F* = 5.40). The SPM{F} showed that different landing actions had significant effects on the gastrocnemius muscle forces at IC-MKF from 0% to 100% (α = 0.05, F* = 4.81). Knee flexion angle, knee valgus angle, knee valgus moment, hamstring muscle forces, and ACL forces had no significant effects (α = 0.05, F*>4.47).

## Discussion

### The influence of different landing test actions on anterior cruciate ligament load

The objective of this study was to investigate the differences and relationships of ACL load, knee angle, joint moment, and muscle forces in three landing test actions. We first hypothesized the differences in the knee angle, moment, and muscle forces of the three test actions. The results of our study partly agree with our hypotheses.

The risk factors for ACL injury are mainly reported in the sagittal plane (Hashemi et al., 2011), such as excessive small knee flexion angle or excessive large knee extension moment. In our study, The ANOVA and SPM also found that differences in the knee flexion angle and knee extension moment.

The ACL load decreases with the increase of knee flexion angle in knee motion (Markolf et al., 1995). Therefore, an increase in the knee flexion angle while landing helps absorb part of the energy and effectively reduces the ACL load, thus reducing the risk of ACL injury (Decker et al., 2003; Kernozek et al., 2005; Cortes et al., 2007). This study found that the ACL load decreased when the knee flexion angle increased during the three landing actions. However, the participants experienced more knee flexion angle in the FVJ group than in the DVJ group, but the FVJ group suffered a higher ACL load than the DVJ group. Previous studies reported that the large extension moment and valgus moments of the landing process might significantly increase the ACL load (Lohmander et al., 2007). In this study, the peak values of knee extension and knee valgus moments of the FVJ group were higher than the DVJ group, which could be one of the reasons for the difference in ACL load in the two landing actions. The FVJ action seems the most applicable to athletes out of three landing test actions (Cortes et al., 2007). However, ACL load in the DL group was the highest compared to the DVJ and FVJ groups, which could be due to the smaller passive knee flexion angle in the IC phase of the DL action compared to the other two actions. As previously reported, ACL load will be effectively reduced when the knee flexion angle exceeds 60°. Hence, the ground reaction force will increase by 68N with each degree reduction of the knee flexion angle (Gerritsen et al., 1995), which could be the reason for the higher ACL load and injury risk of DL action to a certain extent.

Some studies suggested that the risk factors measured in the frontal plane, such as the angle and moment of the knee joint valgus in landing, can cause higher ACL load during the landing process (Bendjaballah et al., 1997; Kar and Quesada, 2012; Kar and Quesada, 2013). The ACL load increases to almost six folds when the valgus angle is 5° (Bendjaballah et al., 1997), and when the angle exceeds 8°, the knee valgus angle poses a significant risk of ACL injury (Kar and Quesada, 2013). However, the maximum knee valgus angle of DL action was approximately 7° during the IC phase. The first peak value of the knee extension moment, knee valgus angle and moment were all within the range of 30% of the IC-MKF phase (Figure 6), which was similar to the phenomenon described by Kar et al. (Decker et al., 2003; Cortes et al., 2007). This phenomenon described that the risk factors measured in the frontal plane, such as valgus angle and moment, also contribute to ACL load, but significantly lower than those measured in the sagittal plane. This study showed that none of the three actions had a maximum of 8° and did not pose a risk of injury to the ACL. Hence, the factors measured in the frontal plane, such as the knee valgus angle and moment, may contribute to the ACL load, but the factors measured in the sagittal plane, such as the knee flexion and extension moment, are the primary risk factors of ACL injury.

### The muscle forces characteristics in different landing actions

The ACL load also depends on muscle forces (Anderson and Pandy, 2003; Hootman et al., 2007). Previous studies reported that the knee extension moment caused by the contraction of the quadriceps femoris could increase the tibial anterior shear force, increasing the ACL load and injury risk (Fagenbaum and Darling, 2003; Alentorn-Geli et al., 2009; Boden et al., 2010). In this study, the quadriceps femoris muscle forces and knee extension moment were highest in the FVJ group and followed by the DVJ and DL groups (Figure 6 and Table 1). The greater knee extension moment and quadriceps femoris muscle forces reported in the FVJ group may be due to the neuromuscular control strategy. The FVJ action needs more distance from the force plates and reaches the maximum vertical jump height immediately after landing Theoretically, the FVJ action should produce larger quadriceps femoris forces, which increases the tibial anterior shear force causing a higher ACL load. Thus, FVJ action may cause a higher ACL injury risk than the other two actions (Fagenbaum and Darling, 2003; Alentorn-Geli et al., 2009; Boden et al., 2010). However, the ACL load of the FVJ action was not the highest in this study. This phenomenon is explicable with the discovery that the ACL load caused by quadriceps femoris tension begins to decrease when the knee flexion angle exceeds 45° and remains little or no impact on ACL load when it exceeds 60° (Arms et al., 1984). According to kinematics results, the knee flexion angle of the three landing actions exceeded 55°; specifically, the FVJ action reached over 60°, which may be why there was no significant difference between the ACL load in the three landing actions during the IC phase.

Further, this may also be related to the contraction of the hamstrings and gastrocnemius muscles. Studies have shown (Bendjaballah et al., 1997) that the posterior shearing force of the tibia due to the contraction of the gastrocnemius and hamstring muscles effectively reduces the load on the ACL when the knee flexion angle is greater than 22°. In our study, the SPM{F} test found differences in gastrocnemius forces between the three actions. DL and DVJ have higher gastrocnemius muscle forces at IC, which provides the ankle joint a greater range of motion to slow the impact of the ground, reducing the risk of ACL injury (Leppänen et al., 2017). The hamstring muscle forces was higher during FVJ maneuvers because FVJ’s larger knee flexion angle increases the activation level of the hamstring muscles (Brazen et al., 2010). Increased knee flexion angle (>30°) on landing attracts more hamstring contraction to participate in coordination (Leppänen et al., 2017), which effectively reduces ACL tension, decreasing the risk of ACL injury. Therefore, a larger knee flexion angle during landing effectively reduces the quadriceps femoris forces. At the same time, it can effectively reduce the ACL load with the coordinated contraction of the hamstring and the gastrocnemius muscles reducing the risk of ACL injury.

### Screening of landing test action

Our second hypothesized that the risk of injury is higher in DL than in DVJ and FVJ. This study showed that the DL action demonstrated a smaller knee flexion angle and greater valgus angle than DVJ and FVJ actions in the IC phase. The relatively unnatural landing action may increase the risk of ACL injury. Therefore, DL action has a higher ACL injury risk than DVJ and FVJ actions, and it is not recommended to use as a test action for ACL injury risk assessment. The FVJ action has higher requirements from the body, which showed a large knee angle, moment and muscle forces during landing, which is more suitable for the actual training and competition conditions. In summary, the FVJ action should be the test action for ACL injury risk assessment. A previous study has also recognized the effectiveness of the FVJ action in ACL injury risk assessment (Cortes et al., 2007).

#### Limitations

Previous studies reported that females have a smaller knee flexion angle than males during the IC phase of the landing (Chappell et al., 2002; Yu et al., 2006). Therefore, using a gender unified landing test is vital in ACL injury risk assessment. However, the difference in the ACL load of three landing test actions was insignificant because the experimental subjects had been professionally trained. Since the subjects in this study were all division II and I basketball and volleyball athletes, the neuromuscular control strategies and landing protection awareness may differ from the normal females. Therefore, a buffered landing strategy may be selected to reduce the risk of ACL injury with unawareness, which can be one of the limitations of this study. Secondly, subjects’ hands were in akimbo during the three landing test actions, which may affect the maximum vertical jump height of DVJ and FVJ action. Nevertheless, this pose unifies the posture during jumping and is widely used during landing tests in previous studies (Meyer and Haut, 2008; Fort-Vanmeerhaeghe et al., 2016).

## Conclusion

Our study indicated minimal differences in knee valgus, knee valgus moment, ACL forces between the three landing actions. However, knee flexion angle, knee extension moments sagittal factors, and quadriceps and gastrocnemius forces are critical factors for ACL injury. This study also revealed that DL action had a significantly smaller knee flexion angle, which may increase the risk of ACL injury, and it is not recommended for risk assessment of ACL injuries. The FVJ action showed a larger knee flexion angle, mobilizing more quadriceps femoris forces, and the moving patterns were relatively similar to the needs of daily training and competition. Therefore, it is recommended to use FVJ action for future studies on ACL injury risk assessment and injury prevention in female college athletes.

## Data Availability

The raw data supporting the conclusions of this article will be made available by the authors, without undue reservation.

## Abbreviations

ACL, Anterior Cruciate Ligament; CMC, Computer Muscle Control; DL, Drop Landing; DOF, Degree of Freedom; DVJ, Drop Vertical Jump; EMG, Electromyography; FD, Forward Dynamics; FVJ, Forward Vertical Jump; IC, Initial Contact; IK, Inverse Kinematics; LESS, Landing Error Scoring System; MKF, Maximum Knee Flexion; RRA, Residual Reduction Algorithms; SPM, Para-metric Mapping; SPM{F}, Para-metric Mapping Characteristics with F Statistics
